# Surface Passivation by Quantum Exclusion: On the Quantum Efficiency and Stability of Delta-Doped CCDs and CMOS Image Sensors in Space

**DOI:** 10.3390/s23249857

**Published:** 2023-12-15

**Authors:** Michael E. Hoenk, April D. Jewell, Gillian Kyne, John Hennessy, Todd Jones, Charles Shapiro, Nathan Bush, Shouleh Nikzad, David Morris, Katherine Lawrie, Jesper Skottfelt

**Affiliations:** 1 Jet Propulsion Laboratory, California Institute of Technology, Pasadena, CA 91109, USA; april.d.jewell@jpl.nasa.gov (A.D.J.); gillian.kyne@jpl.nasa.gov (G.K.); john.j.hennessy@jpl.nasa.gov (J.H.); todd.j.jones@jpl.nasa.gov (T.J.); nathan.bush@jpl.nasa.gov (N.B.); shouleh.nikzad@jpl.nasa.gov (S.N.); 2 Teledyne e2v, Chelmsford CM1 2QU, UK; david.morris@teledyne.com (D.M.); katherine.lawrie@teledyne.com (K.L.); 3 Centre for Electronic Imaging, School of Physical Sciences, The Open University, Milton Keynes MK7 6AA, UK; jesper.skottfelt@open.ac.uk

**Keywords:** CMOS image sensors, delta-doped CCD, radiation damage, stability, image sensor, delta-doped silicon, molecular beam epitaxy

## Abstract

Radiation-induced damage and instabilities in back-illuminated silicon detectors have proved to be challenging in multiple NASA and commercial applications. In this paper, we develop a model of detector quantum efficiency (QE) as a function of Si–SiO_2_ interface and oxide trap densities to analyze the performance of silicon detectors and explore the requirements for stable, radiation-hardened surface passivation. By analyzing QE data acquired before, during, and after, exposure to damaging UV radiation, we explore the physical and chemical mechanisms underlying UV-induced surface damage, variable surface charge, QE, and stability in ion-implanted and delta-doped detectors. Delta-doped CCD and CMOS image sensors are shown to be uniquely hardened against surface damage caused by ionizing radiation, enabling the stability and photometric accuracy required by NASA for exoplanet science and time domain astronomy.

## 1. Introduction

Stable, radiation-hard detectors are essential for ultra-precise photometry in NASA missions planned for the 21st century. The National Academy of Science’s decadal survey, Pathways to Discovery in Astronomy and Astrophysics for the 2020s, highlights NASA’s Transiting Exoplanet Survey Satellite (TESS) Explorer mission for having “ushered in the era of exoplanet science and time-domain astrophysics on a large scale” [1]. NASA and ESA missions, such as Kepler, TESS, GAIA, and Euclid, are testaments to the advances in silicon detector sensitivity and stability over the last 50 years. Despite these advances, recent observations of charge-coupled device (CCD) instabilities caused by ultraviolet (UV) illumination raise important questions about the stability and photometric accuracy of silicon detectors in space [2].

Detector stability has been a long-standing challenge in space and commercial applications. In 1984, NASA discovered quantum efficiency hysteresis (QEH) in charge-coupled devices (CCDs) on Hubble Space Telescope’s Wide Field and Planetary Camera (WF/PC) [3]. QEH is characterized by variations in quantum efficiency with exposure levels, caused by uncontrolled charging of defects in the illuminated CCD surface [4]. The technique chosen to eliminate QEH in WF/PC-1 CCDs was a UV flood, in which the detectors were periodically exposed to solar UV radiation to generate a negative charge on the detector surface. Meanwhile, JPL developed and tested several new backside charging processes to enable long-term stability and eliminate QEH in WF/PC-2 CCDs without requiring a UV flood, including the platinum flash gate [5,6,7]. Backside charging methods developed after WF/PC include chemisorption charging [8] and atomic layer deposition of negatively charged aluminum oxide [9]. Radiation tests undertaken for the Extreme Ultraviolet (EUV) Variability Experiment showed that the chemisorption charging process is not stable against EUV radiation, while CCDs passivated by surface doping survived the full lifetime EUV exposure; moreover, surface passivation using MBE growth proved to be far more effective than ion implantation in stabilizing detector QE against EUV-induced surface damage and charging [10].

Surface doping by ion implantation and annealing is currently the leading commercially available method for the surface passivation of silicon detectors in space. Degradation of QE and stability caused by ionizing radiation is a concern for these detectors, for reasons that we will explore in this paper. In some cases, the degradation can be severe. In 1995, ESA and NASA launched the Solar and Heliospheric Observatory (SOHO), with an Extreme Ultraviolet Imaging Telescope (EIT). Soon after operations began, EIT suffered a significant loss of signal caused by EUV-induced surface damage in the CCD [11,12,13]. In light of the damage and instabilities in the SOHO EIT CCD, e2v and Lockheed Martin’s Solar and Astrophysics Laboratory began developing improved surface passivation processes for CCDs in the GOES N and O solar X-ray instruments [14,15,16]. The resulting UV-enhanced CCDs are passivated with an ultra-shallow ion-implantation and laser anneal process. Radiation tests showed a linear drop in response with increasing solar X-ray exposure, indicating that the UV-enhanced, ion-implanted CCD surface is not completely passivated against surface damage and charging [15]. UV-enhanced CCDs were found to exhibit low-level QEH in ground tests and in space. In 2009, residual QEH was discovered in ground tests of CCDs developed for Hubble Space Telescope’s Wide Field Camera 3 (WFC3) [17]. To mitigate QEH on orbit, WFC3 CCDs are periodically subjected to a pinning exposure after each annealing cycle [18]. In 2013, European Southern Observatory (ESO) astronomers reported that Janesick’s UV-flood process can improve the UV QE of ion-implanted detectors by up to 50% in ground-based telescopes [19].

JPL pioneered the use of low temperature molecular beam epitaxy (MBE) to passivate back-illuminated CCDs, enable near 100% internal QE and long-term stability, and eliminate the QEH problems observed in WF/PC CCDs [20,21]. MBE growth enables atomic-layer control over the dopant profile, while delta doping pushes dopant densities to their limits by transitioning from 3D to 2D dopant profiles. In conventional 3D doping methods, dopant atoms are randomly distributed in the silicon lattice, and the density of electrically active dopants is constrained by clustering and solid solubility limits of dopants in silicon. MBE growth of 2D-doped silicon breaks through the electrical saturation barrier inherent in 3D doping processes [22]. The growth process involves closing the silicon shutter to halt the deposition of silicon atoms, depositing dopant atoms on the atomically clean silicon surface, and resuming silicon growth to encapsulate and stabilize the 2D-doped layer. Under suitable conditions of ultra-high vacuum and substrate temperature, the dopant atoms form a self-organized two-dimensional (2D) surface phase, enabling the incorporation of electrically active dopant atoms at concentrations as high as half a monolayer. JPL’s delta doping process uses a sheet density of 2 × 10^14^ cm^−2^, comprising approximately 30% of a monolayer in (100) silicon. The 2D doping process is commonly called delta doping because the dopant profile resembles the Dirac delta function. JPL later improved the process by growing multiple, stacked delta-doped layers to form a 2D-doped superlattice, which enhances both the stability and surface conductivity of delta-doped detectors in high-radiation environments [23,24]. Passivation of CCDs and CMOS image sensors using these 2D doping methods enables near-100% internal quantum efficiency (IQE) with exceptional stability against UV-induced surface damage and charging.

In this paper, we develop a QE model and apply the model to analyze UV-induced degradation and instabilities in silicon detectors passivated using surface doping methods. Surface passivation has been a long-standing problem for silicon detectors in space because of the connections between radiation-induced surface damage, surface charge and QE degradation. By developing a model of QE as a function of surface and interface trap densities and comparing the results with published data on UV-induced surface damage and QE instabilities in silicon detectors, we show that state-of-the-art ion-implanted detectors are sensitive to surface charge variability at a level of 10^11^ cm^−2^, whereas delta-doped detectors remain stable to variable surface charge and trap densities at levels as high as 10^14^ cm^−2^. The stability of delta-doped detectors against high levels of UV-induced surface damage was demonstrated experimentally in accelerated lifetime tests in which delta-doped CMOS image sensors were exposed to pulsed deep ultraviolet lasers over a period of several months [25]. We explore the reasons for this stability and its implications for precision photometry in space.

## 2. Materials and Methods

### 2.1. Overview

The QE model developed here follows the ion-implanted CCD model in Stern et al. 1994 [26], with new modifications and additions to study QE and stability of radiation-damaged detectors. The essential new aspect of this model is the parameterization of surface charge in terms of interface and oxide trap densities ($N_{it}$ and $N_{ot}$), which enables a new interpretation of previously published QE and stability data from ion-implanted and delta-doped detectors. The required additions to previous models include self-consistent solutions of Poisson’s equation to calculate the surface charge, electric field, and potential in the surface depletion region, and using Shockley-Read-Hall theory and $P_{b0}$ trap parameters (cross-section and density of states) to calculate the surface recombination velocity. Calculations are carried out using $N_{it}$ and $N_{ot}$ as independent variables, and the results are compared with published QE data to estimate trap densities and explore the causes and implications of time-variable surface charge and QE.

### 2.2. Minority Carrier Transport in Heavily Doped Silicon

The physics of minority carrier transport in degenerately doped silicon plays an important role in our QE calculations. Historically, studies of bipolar transistor performance uncovered systematic deviations between the data and models, which led to the discovery that the minority carrier density in degenerately doped silicon is higher than expected. To model this effect, minority carrier concentration in degenerately doped silicon is parameterized in terms of an apparent bandgap narrowing, $\Delta E_{g}(N_{A})$, which obeys the modified mass-action law [27,28,29,30]:

(1) $$n_{0}p_{0}=n_{io}^{2}(T)\cdot exp\left(\frac{\Delta E_{g}(N_{A})}{kT}\right)$$

where $n_{0}$ and $p_{0}$ are the equilibrium electron and hole concentrations, $N_{A}$ is the acceptor concentration in the p+ doped surface, and $n_{io}$ is the intrinsic carrier concentration in undoped silicon. In this paper, we follow Stern et al. in using empirical formulae for the apparent bandgap narrowing, $∆E_{g}\left(N_{A}\right)$, the electron mobility, $\mu_{n}(N_{A})$, and the electron lifetime, $\tau_{n}(N_{A})$, in degenerately doped p+ silicon [26,27,28]:

(2) $$∆E_{g}\left(N_{A}\right)=(9\;meV)\cdot \left(F(N_{A})+\sqrt{{F(N_{A})}^{2}+0.5}\right)$$

(3) F(NA)=ln⁡NA1017

(4) $$\mu_{n}(N_{A})=232+\frac{1180}{\left(1+{\left(\frac{N_{A}}{8\times 10^{16}}\right)}^{0.9}\right)}\;{cm}^{2}V^{-1}s^{-1}$$

(5) $$\tau_{n}^{-1}(N_{A})=\left(3.45\times 10^{-12}N_{A}+0.95\times 10^{-31}N_{A}^{2}\right){\;s}^{-1}$$

The equilibrium concentration of electrons in p-type silicon is given by Boltzman statistics [31]:

(6) $$n_{0}=N_{c}(T)\cdot \exp\left(\frac{{-E}_{c}}{kT}\right)$$

where $N_{c}(T)$ is the density of states in the silicon conduction band, and, for simplicity, the Fermi energy has been set to zero (i.e., all energies in this model are measured relative to a constant Fermi level). From Equations (1) and (6), we can derive the following relationship between the conduction band energy and the surface dopant concentration:

(7) $$E_{C}(x)=kT\cdot ln\left(\frac{N_{A}(x){\cdot N}_{c}(T)}{n_{io}^{2}(T)}\right)-\Delta E_{g}(N_{A}(x))$$

in which we have implicitly assumed full activation of the dopants in degenerately doped silicon. The important consequence of Equation (7) is that bandgap narrowing broadens the surface barrier and reduces the near-surface electric field created by ultra-shallow ion implantation. As we shall see, bandgap narrowing has a significant effect on the QE and stability of ion-implanted silicon detectors. Delta-doped detectors don’t suffer from this limitation because MBE growth produces an ultrathin surface passivation layer, characterized by a peak dopant density that is two orders of magnitude higher than ion implantation and an abrupt junction at the MBE-detector interface.

### 2.3. Si–SiO_2_ Interface and Oxide Traps

Defects in silicon surfaces and oxides, including the well-known $P_{b0}$ and $P_{b1}$ defects [32] and the *E*’ center in SiO_2_ [33], are inherent in the microscopic structure of silicon detectors. For the purposes of this model, surface defects are parameterized by the densities of interface traps, $N_{it}$, and oxide charge, $N_{ot}$. Trapping and detrapping of charge in these defects depend on both the environment and the trap location relative to the Si–SiO_2_ interface.

Oxide traps (sometimes called slow traps) are identified with ‘fixed’ oxide charge, meaning that the charge state of the trap does not immediately respond to changes in the surface potential. Oxide charge changes over time, especially when the surface is damaged by exposure to ionizing radiation (e.g., high-energy photons) or stressed by exposure to hot carriers (i.e., damage and charging of SiO_2_ under charge injection stress).

Interface traps (also called fast traps) respond to variations in the surface potential by changing the density of trapped charge. Surface charge density calculations require an integration over the silicon bandgap of the product of the density of states in the silicon bandgap ($D_{it}dE$) and the Fermi function, in which donor-like states occupy the lower half of the bandgap, and acceptor-like states occupy the upper half. Surface recombination in silicon detectors is dominated by $P_{b0}$ traps, and we have used published data to model the density of states [34] and cross-section [35] of $P_{b0}$ traps at the Si–SiO_2_ interface. The unknown parameter in these calculations is the surface potential relative to the Fermi level, which must be calculated by solving Poisson’s equation.

### 2.4. Poisson’s Equation, Surface Depletion, and the Surface Potential

Surface charge lies at the heart of the problem that we are trying to solve, insofar as detector instabilities may be traced to the time-variable occupation of interface and oxide traps. The calculation of the surface charge in terms of $N_{it}$ and $N_{ot}$ entails a self-consistent solution of Poisson’s equation in the near-surface space charge region (either depletion or accumulation, depending on the polarities of surface charge and surface doping). We begin by guessing the surface potential relative to the Fermi energy and calculating the density of surface charge based on this guess. We can then solve Poisson’s equation to calculate the electric field and potential in the surface depletion region, accounting for space charge due to ionized acceptors and residual carrier densities. The net charge is calculated as the sum of charge densities in the oxide and interface traps and the integrated space charge in the surface depletion region. To arrive at a self-consistent solution of Poisson’s equation, the surface potential is iterated until the net charge is zero. This calculation provides essential parameters for the silicon band structure as a function of depth in the detector, with $N_{it}$ and $N_{ot}$ as the independent variables. In particular, we now know the conduction band energy, $E_{c}(x)$, and the electric field, $E\left(x\right)$, at thermal equilibrium.

### 2.5. Photogenerated Charge and Currents in Illuminated Detectors

Illumination generates non-equilibrium charge distributions and currents in detectors. Under steady-state illumination, the generation and recombination rates as a function of depth from the surface are given by:

(8) $$G_{n}\left(x,\lambda \right)=\phi_{0}\cdot \alpha (\lambda )\cdot \exp(-\alpha (\lambda )\cdot x)$$

(9) $$U_{n}(x)=\frac{n\left(x\right)-n_{0}\left(x\right)}{\tau_{n}(x)}$$

where $G_{n}\left(x,\lambda \right)$ and $U_{n}(x)$ are the position-dependent rates of generation and recombination of minority carriers, $\phi_{0}$ is the photon flux at wavelength λ, α(λ) is the wavelength-dependent coefficient of absorption in silicon [36], and $n\left(x\right)-n_{0}\left(x\right)$ is the excess minority carrier concentration relative to thermal equilibrium. In order to calculate the minority carrier density and current in an illuminated detector, we solve the coupled current and continuity equations using the small-signal approximation [31]:

(10) $$J_{n}\left(x\right)=q{\cdot \mu }_{n}\cdot n\left(x\right)\cdot E\left(x\right)+q{\cdot D}_{n}\cdot \frac{\partial }{\partial x}n(x)$$

(11) $$\frac{1}{q}\cdot \frac{\partial }{\partial x}J_{n}\left(x\right)=-G_{n}\left(x,\lambda \right)+U_{n}(x)$$

where $J_{n}\left(x\right)$ is the electron current in the illuminated detector, $n\left(x\right)$ is the (non-equilibrium) electron density, and $E\left(x\right)$ is the electric field.

The silicon material parameters governing charge transport in degenerately-doped silicon are the electron mobility ($\mu_{n}(N_{A})$ in Equation (4)), the minority carrier lifetime ($\tau_{n}(N_{A})$ in Equation (5)), and the diffusion coefficient (calculated with the Einstein relation, $D_{n}(N_{A})=\mu_{n}(N_{A})\cdot kT$). Because these parameters are inextricably linked in empirical models of bandgap narrowing, it is important that the parameter models are consistent with the model used to calculate bandgap narrowing. All of these parameters depend on the surface dopant profile, $N_{A}(x)$, which must be known in order to carry out the calculations.

To solve these equations, we subdivide the detector into *N* regions according to depth from the surface (*x*_0_, *x*_1_, …, *x_N_*), and assume that the dopant density and electric field are constant in each region. With these assumptions, Equations (8)–(11) have closed form solutions within each region, and we can use a generalization of Blouke’s CCD model to ensure continuity of *n*(*x*) and *J*(*x*) at the *N* − 1 boundaries between regions [37]. Boundary conditions appropriate to a back-illuminated silicon detector are as follows:

(12) $$J_{n}\left(x_{0}\right)=q\cdot S\cdot \left[n\left(x_{0}\right)-n_{0}\left(x_{0}\right)\right]$$

(13) $$n\left(x_{N}\right)-n_{0}\left(x_{N}\right)=0$$

Equation (12), the boundary condition at the illuminated surface (${x=x}_{0}$), represents surface recombination due to traps at the Si–SiO_2_ interface. The minority carrier current at the surface is proportional to the excess minority carrier density, and the proportionality constant, *S*, is the surface recombination velocity (Section 2.6). Equation (13), the boundary condition at the edge of the detector collection well (${x=x}_{N})$, has essentially the same form as Equation (12), except that the proportionality constant is taken to be infinite, which is equivalent to the assumption that all excess minority carriers at the right boundary are captured by the detector’s charge collection well.

### 2.6. Shockley–Read–Hall Theory and the Surface Recombination Velocity

The surface recombination velocity (*S* in Equation (12)) dominates the detector QE in the UV and EUV regions of the spectrum. The dependence of the surface recombination velocity on the trap cross-section, $\sigma_{p}$ and $\sigma_{n}$, and energy level, $E_{t}-E_{i}$, is given by Shockley–Read–Hall (SRH) formalism [38]:

(14) $$S\equiv \frac{\sigma_{p}\sigma_{n}v_{th}\cdot p_{s}\cdot N_{it}}{\sigma_{n}\left[n_{s}+n_{i}\exp\left(\frac{E_{t}-E_{i}}{kT}\right)\right]+\sigma_{p}\left[p_{s}+n_{i}\exp\left(\frac{E_{t}-E_{i}}{kT}\right)\right]}$$

The SRH theory has been further refined in models of solar cell performance by replacing $N_{it}$ in Equation (14) with a continuous density of states, $D_{it}\left(E_{t}-E_{i}\right)dE$, and integrating over the silicon bandgap [39]. This formalism is used to calculate the surface recombination velocity in this paper. As described in Section 2.3, the density of states and cross-sections of $P_{b0}$ traps were taken from published data [34,35].

The surface densities of electrons and holes, $n_{s}$ and $p_{s}$, are calculated based on the surface potential, $E_{c}(x_{0})$, which depends on the surface charge and is derived by solving Poisson’s equation (see Section 2.4). The surface electron density can be calculated using Equation (6), and the surface hole density can be calculated using Equation (1) based on the additional assumption that the product of the minority and majority carrier densities is constant in the surface depletion region. This entails an approximation in which bulk recombination (primarily Auger recombination in the heavily doped silicon surface) is assumed to be negligible in the space charge region. The surface depletion region is quite narrow in ion-implanted detectors, and in any case surface recombination is dominated by interactions of photogenerated charge with Si–SiO_2_ interface traps.

The surface recombination velocity and surface electron density are extremely sensitive to the surface potential, which means that these calculations must be repeated for each value of the independent variables (i.e., the densities of interface traps, $N_{it}$, and oxide charge, $N_{ot}$). Stern’s model simplifies the calculations by parameterizing the surface using a single parameter, the effective surface recombination velocity, which is defined in terms of the carrier concentrations at a fictitious surface located at the edge of the surface depletion region in the detector, $x=x_{d}$.

(15) $$J_{n}\left(x_{d}\right)=q\cdot S_{eff}\cdot \left[n\left(x_{d}\right)-n_{0}\left(x_{d}\right)\right]$$

(16) $$S_{eff}\approx S_{0}\exp\left(\frac{∆E}{kT}\right)$$

In Equation (16), $S_{0}$ is the surface recombination velocity in the absence of surface charge, and ∆E is the magnitude of band bending caused by surface charge. Stern’s approach avoids the necessity of solving Poisson’s equation but offers little insight into the quantitative relationships between surface charge, trap densities, QE, and stability in silicon detectors that are explored in this paper. Nevertheless, Equation (16) highlights the exponential dependence of surface recombination on the surface potential, which is why relatively small changes in surface charge can have a significant effect on the QE and stability of ion-implanted detectors. These relationships are explored quantitatively in Section 3. As a final comment, we note that ∆E is positive for surface depletion, and negative for accumulation, which is why high QE is easier to achieve with backside charging than surface doping, whereas surface doping provides better stability and a longer lifetime in a radiation environment [10]. As we shall show, delta-doping is unique in providing both high QE and exceptional stability against radiation-induced surface damage.

### 2.7. Quantum Efficiency

After solving the current and continuity equations to obtain the minority carrier current and charge density ($J_{n}\left(x\right)$ and $n\left(x\right)$), the QE at wavelength λ can be calculated according to the formula:

(17) $$QE\left(\lambda \right)=\frac{T\left(\lambda \right)}{\phi_{0}}\cdot \left\{J_{n}\left(x_{N}\right)-J_{n,\;dark}\left(x_{N}\right)+\phi_{0}\cdot \left[\left(\exp(-\alpha \cdot x_{N}\right)-\exp\left(-\alpha \cdot x_{W}\right)\right]\right\}$$

The function T(λ) is the transmittivity of the surface, which accounts for reflection and absorption losses in the oxide. By setting T(λ)=1, this formula can also be used to calculate the internal quantum efficiency (IQE). The flux, $\phi_{0}$, represents the rate at which photons enter the detector at the surface ($x=x_{0}$) after accounting for reflection and absorption losses. The net current, $J_{n}\left(x_{N}\right)-J_{n,\;dark}\left(x_{N}\right)$, is the detector signal corrected for dark current, which is calculated by solving Equations (8)–(11) in the dark ($\phi_{0}=0$). The third term corresponds to photon absorption in the collection well (between $x_{N}$ and $x_{W}$), which becomes important in the near infrared and soft X-ray spectral ranges, where the photon absorption length approaches the thickness of the silicon detector.

## 3. Results

### 3.1. Quantum Efficiency of Ion-Implanted and Delta-Doped Detectors

**Figure 1:**
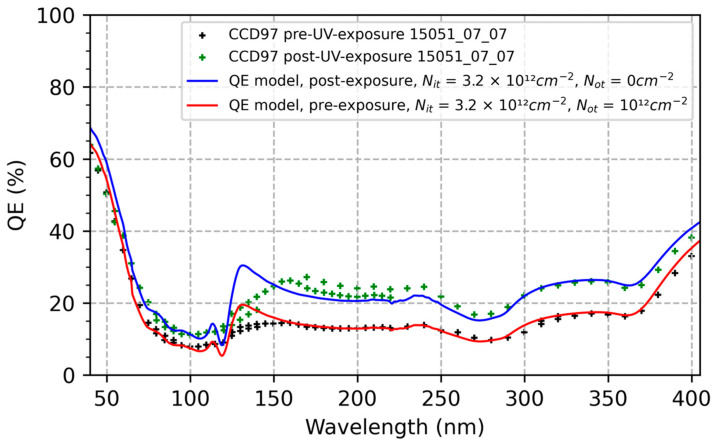
QE measurements of ion-implanted CCDs before and after prolonged exposure to 200 nm photons show significant UV-induced enhancement [40]. Model calculations show that the observed QE enhancement is consistent with UV-induced neutralization of oxide charge at a level of $10^{12}\;{\mathrm{cm}}^{-2}$.

**Figure 2:**
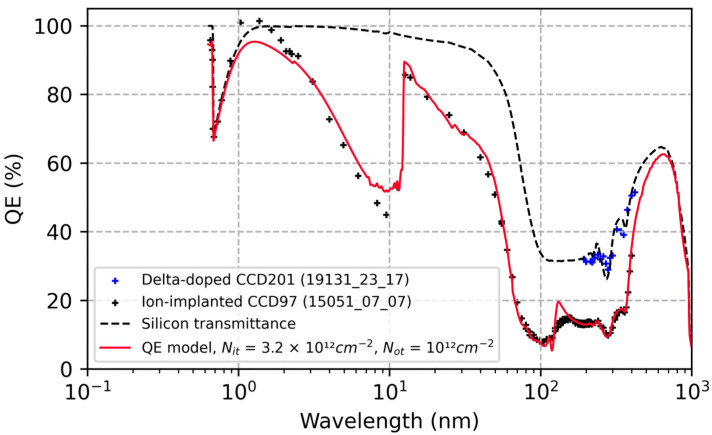
QE measurements of the ion-implanted CCD in Figure 1 are compared with the model over the EUV to visible spectral range. The QE data are consistent with model calculations for a combined interface and oxide charge density of $\sim 4\times 10^{12}\;{\mathrm{cm}}^{-2}$. For comparison, delta-doped CCD QE data are plotted over the same spectral range, along with the reflection-limited QE (silicon transmittance). All QE measurements were performed by Open University [40,41,42].

Heymes et al. observed a roughly 50% increase in the QE of ion-implanted CCD97 detectors after prolonged exposure to 200 nm photons (Figure 1) [40]. The observed UV-induced QE enhancement saturated after two hours of exposure and remained relatively stable through another 15 h of continuous exposure. In Figure 2, we’ve plotted the QE of the same ion-implanted detector over a spectral range spanning extreme to near UV wavelengths. For comparison, we’ve plotted the reflection-limited QE of silicon detectors over this spectral range, together with the QE of a delta-doped CCD201 detector measured at Open University [41]. The delta-doped CCDs used in these experiments were developed in a collaboration between JPL and Teledyne e2v for the qualification of high-performance UV detectors for spaceflight. In this paper, comparisons between QE data and models are used to provide quantitative estimates of the sensitivity of ion-implanted and delta-doped detectors to surface charge, and to explore the physical and chemical mechanisms underlying UV-induced surface damage and degradation of detector QE and stability.

### 3.2. Ion-Implanted Detectors: QE and Stability vs. Surface Charge

The QE of the ion-implanted detector in Figure 1 and Figure 2 is strongly dependent on surface charge. Consultations with Teledyne e2v revealed that the CCDs tested by Heymes et al. [40] are not representative of current device capabilities, and more recent devices are expected to have improved QE and stability. Figure 1 shows that the trap densities in the CCDs tested by Heymes et al. are relatively high, which may have been caused by exposure to EUV and soft X-ray radiation during prior experiments at the BESSY II synchrotron [42]. Further study is needed to validate these results with more representative devices. We also note that surface charging effects depend on experimental conditions, and care is needed when comparing data from different sources. Heymes’ UV-flood experiments were performed by exposing the detector to 200 nm photons while the detector was cold and under vacuum. The UV flood developed for WF/PC 1 is performed when the detector is near room temperature and requires oxygen to enable UV-catalyzed chemisorption of oxygen ions on the detector surface. The WFC3 QE-pinning process uses the calibration lamp to flood the detectors with visible light at photon energies below the threshold for hot carrier injection into the oxide.

**Figure 3:**
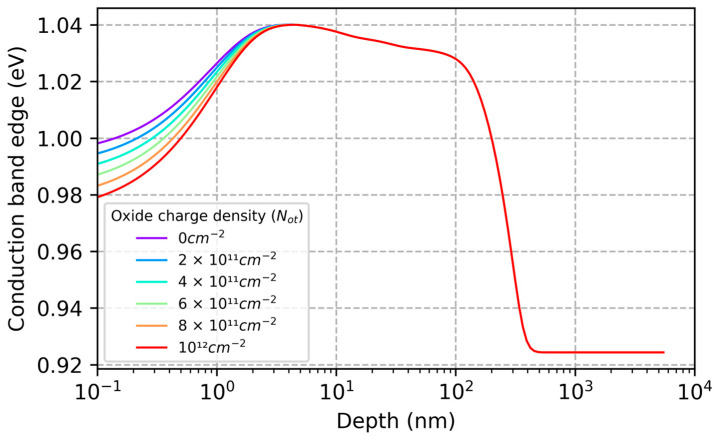
The surface potential in the ion-implanted detector in Figure 1 ($N_{it}=3.2\times 10^{12}\;{\mathrm{cm}}^{-2}$) only changes by about 20 meV as the oxide charge density (*N_ot_*) varies from 0 to $10^{12}\;{\mathrm{cm}}^{-2}$. Despite the ultra-shallow dopant distribution in the ion-implanted detector, model calculations show that the width of the surface potential barrier exceeds 100 nm. Because the surface barrier is so wide, electrons generated in the tail of the implant can interact with surface traps, which ultimately limits the QE and stability that can be achieved in ion-implanted detectors.

**Figure 4:**
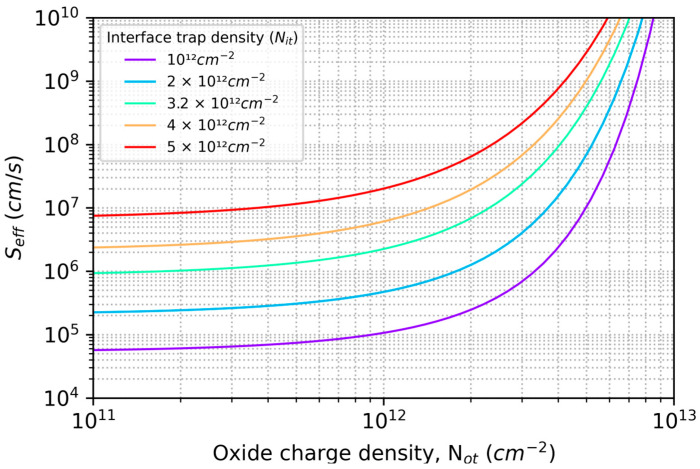
The effective surface recombination velocity (*S_eff_*) is plotted as a function of oxide charge density for various interface trap densities (*N_it_*). The strong dependence of *S_eff_* on surface charge elucidates the connection between seemingly small changes in surface potential (Figure 3) and observations of large UV-induced changes in the QE of ion-implanted CCDs (Figure 1, Figure 5 and Figure 6).

**Figure 5:**
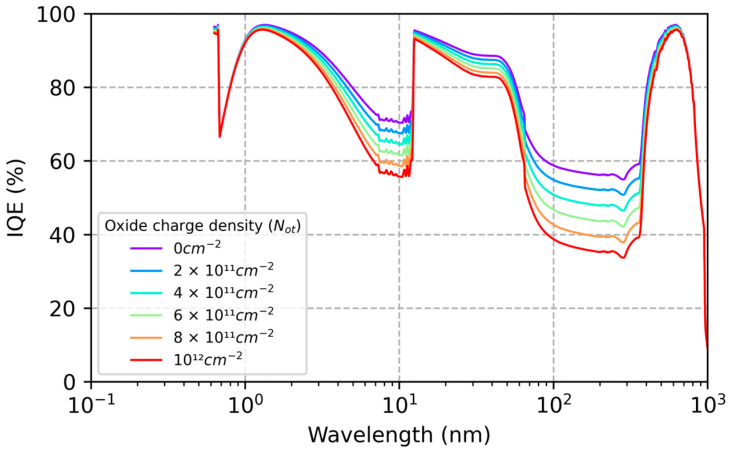
The diffusion-drift QE model was used to calculate the internal quantum efficiency (IQE) of the ion-implanted detector in Figure 1 and Figure 2 as a function of oxide charge density. The UV-induced QE enhancement observed by Heymes et al. [40] (Figure 1) corresponds to large changes in IQE (from approximately 35% to 55% at 280 nm) as *N_ot_* varies over a range of $10^{12}\;{\mathrm{cm}}^{-2}$. Significant variations in IQE are seen in response to changes in surface charge density as small as $10^{11}\;{\mathrm{cm}}^{-2}$.

**Figure 6:**
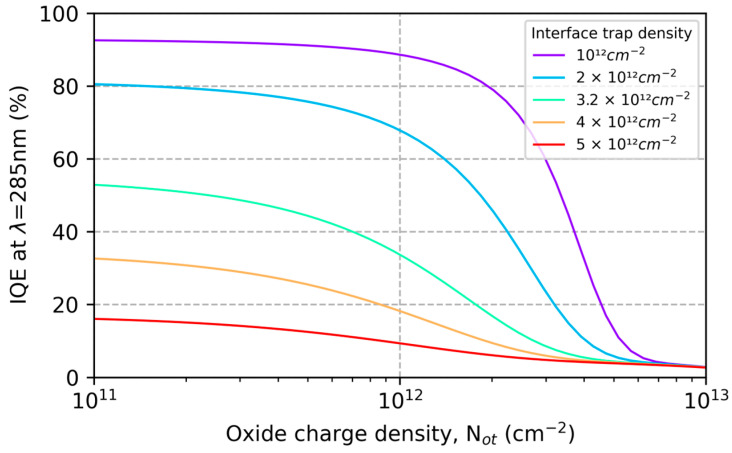
Accumulated damage from ionizing radiation causes an overall reduction in QE, and also leads to instabilities, illustrated here by plotting internal quantum efficiency (IQE) as a function of the oxide charge density, $N_{ot}$. The curve with $N_{it}=3.2\times 10^{12\;}{\mathrm{cm}}^{-2}$ corresponds to the CCD in Figure 1, which is shown to be sensitive to variations in surface charge as small as $10^{11}\;{\mathrm{cm}}^{-2}$.

By varying the model parameters ($N_{it}$ and $N_{ot}$) and comparing the results with QE data in Figure 1 and Figure 2, we can begin to explore the causes of ion-implanted detector inefficiencies and instabilities in terms of surface charge and minority carrier transport in degenerately doped silicon surfaces. Figure 3 plots the changes in the conduction band energy near the surface of this ion-implanted CCD as the oxide charge density varies from 0 to $10^{12}\;{cm}^{-2}$. From this we can conclude that the QE enhancement observed in Heymes et al. [40] corresponds to variations in the surface potential of only 20 meV (Figure 3). The sensitivity of the surface recombination velocity to interface and oxide trap densities are shown in Figure 4, which graphically illustrates the exponential sensitivity on surface potential expressed in Equation (16). Continuing this analysis, Figure 5 shows the internal QE (IQE) of the ion-implanted CCD as a function of oxide charnge density ($N_{ot}$), while Figure 6 focuses on the internal QE at λ=285 nm, where the silicon absorption length approaches its minimum of 4 nm. The trap densities used in these calculations are based on comparisons of data and models shown in Figure 1 and Figure 2. From this analysis, we can conclude that the CCD QE reported by Heymes et al. is sensitive to variations in surface charge density on the order of $10^{11}\;{cm}^{-2}$. Viewed in light of Figure 4 and Equation (16), we can infer that the QE and stability of ion-implanted detectors are interrelated in radiation-damaged detectors, and the rate of surface recombination grows exponentially worse as the density of surface traps accumulates over time.

As expected, the greatest variability in IQE of an ion-implanted detector is seen in the UV spectral range, where absorption takes place near the surface (see Figure 5). However, significant changes are seen across the entire spectral range from soft X-rays to visible wavelengths, offering additional insights into the underlying physics of minority carrier charge transport in ion-implanted detectors. Whereas the high surface dopant density created by ion implantation limits the depth of the surface depletion region to approximately 3nm (see Figure 3), the QE data show that minority carriers generated at much greater depths are being lost to surface recombination. The worst-case IQE is 35% at λ=285 nm, where the photon absorption length in silicon is near the minimum value of 4 nm (Figure 5 and Figure 6). Surprisingly, the IQE is still below 60% at λ=10 nm, where the absorption length in silicon is close to 40 nm (Figure 5). The low IQE measured at this EUV wavelength suggests that a significant fraction of photoelectrons generated at depths far beyond the surface depletion layer are lost to surface recombination. This can be understood by studying the conduction band vs. depth profile in the ion implanted surface (Figure 3), which shows that there is virtually no barrier preventing electrons generated at depths up to 100 nm from interacting with surface traps. This rather surprising result is a consequence of bandgap narrowing in degenerately doped silicon, as described in Section 2. Bandgap narrowing therefore limits the QE and stability of ion-implanted CCDs and CMOS image sensors. This limitation can be overcome by passivating the detector surface with delta-doped superlattices developed at JPL, which produce a surface barrier that is only a few nanometers in width (see Section 3.3).

### 3.3. Delta-Doped Detectors

JPL demonstrated stable, near-100% internal QE in delta-doped CCDs in the 1990s [21,22]. The unique stability of delta-doped detectors against high levels of radiation-induced surface damage was demonstrated experimentally in accelerated lifetime tests performed by Alacron and Applied Materials in 2012–2013. In these tests, CMOS image sensors passivated with a delta-doped superlattice were periodically measured during months-long, continuous exposure to high-intensity, pulsed, deep UV lasers at 193nm and 263 nm. Surface damage levels under these conditions were measured to be in the range of 10^14^ cm^−2^, and yet characterization of the detectors before, during, and after exposure showed that the QE remained stable to within 1% [25,41]. The uniqueness of this stability is highlighted by independent studies of the degradation behavior and damage mechanisms in CCD image sensors exposed to deep UV radiation [43,44]. In this section, we explore the physical and chemical reasons for the unique QE and radiation hardness of delta-doped detectors and surfaces.

#### 3.3.1. Interface Trap Density in Delta-Doped Detectors

One of the motivations for JPL’s development of delta-doped superlattices was to counteract the surprisingly low surface conductivity of detectors passivated with a single delta-doped layer [25]. The low conductivity of delta-doped surfaces is caused by the immobilization of minority carriers by traps at the Si–SiO_2_ interface. The existence of surface charge densities approaching the sheet density of dopant atoms in the delta layer has been established experimentally by studies of electron transport in delta-doped surfaces [45], and by measurements of the conductivity of superlattice-doped surfaces before and after exposure to pulsed DUV lasers [25]. Clark et al. measured the effects of surface proximity on electron transport in ultra-shallow delta-doped layers with a sheet density of 1.7 × 10^14^ cm^−2^ and discovered that delta-doped surfaces are insulating when encapsulated by less than 3 nm of silicon. JPL measured the surface conductivity of superlattice-doped silicon wafers before and after exposure to DUV lasers to estimate the interface trap densities created during the accelerated lifetime tests cited above. Based on these measurements, the density of interface traps is on the order of 10^14^ cm^−2^ prior to DUV exposure, and increases by as much as 10^14^ cm^−2^ as a result of DUV-induced surface damage (see Figure 6 in Reference [25]). For reference, each layer in a delta-doped superlattice contains approximately 2 × 10^14^ dopants/cm^2^, which corresponds to ~30% of a monolayer in (100) silicon. The high surface charge density in delta-doped detectors may be explained by the magnitude of the electric field at the Si-SiO_2_ interface. According to our model, the surface electric field is on the order of 1 V/nm, which is sufficient to induce dielectric breakdown in the oxide.

#### 3.3.2. Radiation Hardness of Delta-Doped Detectors

**Figure 7:**
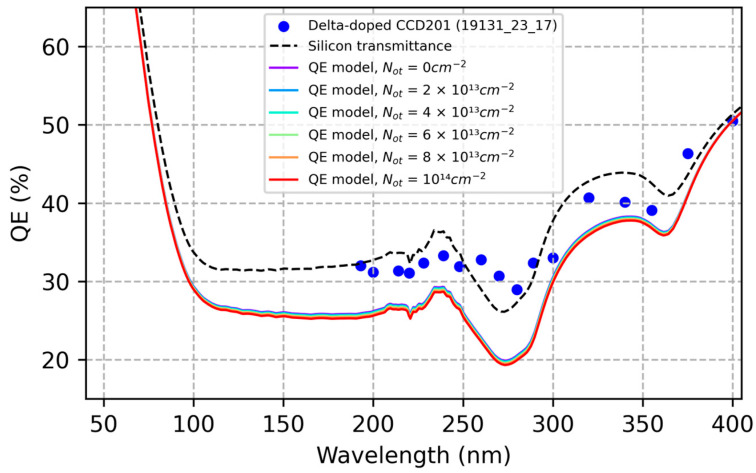
QE model calculations predict exceptional stability against UV-induced surface damage in delta-doped detectors. According to the model, the QE remains stable to within 1% for oxide charge densities ranging from 0 to 10^14^ cm^−2^, in agreement with published data on accelerated lifetime tests of delta-doped CMOS image sensors [25]. For comparison, we have plotted the measured QE of a delta-doped CCD (blue circles), together with the reflection-limited QE (silicon transmittance) of an uncoated silicon detector (dashed line). Differences between the measured and calculated QE are attributed to quantum transport in the MBE-grown silicon layer (see Section 3.3.3). QE data in this Figure were published in Reference [41] and are also plotted in Figure 2.

The stability and radiation hardness of delta-doped detectors were investigated by applying the diffusion-drift QE model to delta-doped detectors. Based on the interface trap densities described in Section 3.3.1, we calculated the QE of a delta-doped detector with an interface trap density of 10^14^ cm^−2^ and oxide charge densities ranging from 0 to 10^14^ cm^−2^ (Figure 7). The model predicts that the QE of a delta-doped detector is stable to within 1% despite variations in surface charge density as large as 10^14^ cm^−2^. Whereas this stability agrees with data from the accelerated lifetime tests cited above, the calculated QE is significantly lower than the measured QE shown in Figure 7. This discrepancy represents a limitation of the diffusion-drift QE model, which is discussed in Section 3.3.3.

**Figure 8:**
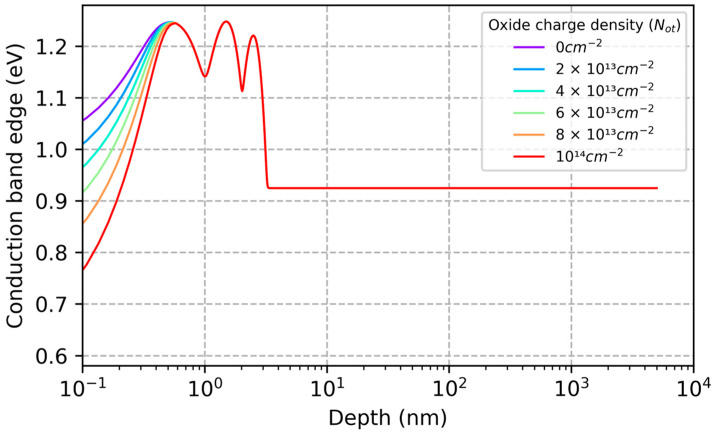
The conduction band energy vs. depth is plotted for a delta-doped detector with oxide charge densities in the range of 0 to 10^14^ cm^−2^. The depletion depth in the MBE-grown silicon is effectively pinned at the location of the delta layer nearest the surface. The stability of the depletion depth is one of the factors in the QE stability of delta-doped detectors. The other factor is the ultrathin surface passivation layer created by MBE growth (see Figure 3 for comparison).

To understand the reasons for the stability in calculated QE (Figure 7), we studied the dependencies of surface depletion and recombination on oxide charge density. Figure 8 shows that the depth of the surface depletion layer is pinned at the location of the nearest delta-doped layer, despite variations in surface charge density as large as 10^14^ cm^−2^. This is a necessary but insufficient condition for QE stability, as can be seen by comparing with Figure 3 and Figure 8. In the case of ion-implanted detectors, the depletion layer depth remains stable over the relevant range of surface charge, but the QE is very sensitive to variable surface charge (Figure 1 and Figure 5). To understand the stability of delta-doped detectors, we must also consider the effective surface recombination velocity, which depends exponentially on the surface potential (Equation (16)). The calculated effective surface recombination velocity in a delta-doped detector varies from 1.5 × 10^12^ cm/s at *N_ot_* = 0, to 3 × 10^18^ cm/s at *N_ot_* = 10^14^ cm^−2^. The significance of this result is that the surface recombination velocity is effectively infinite, and the surface boundary condition in a delta-doped detector consequently takes the form of Equation (13). This means that the QE calculated using the drift-diffusion model does not depend on the surface recombination velocity. In short, the diffusion-drift model predicts that delta-doped detectors are stable against high levels of radiation-induced surface damage because all excess carriers at the surface are lost to recombination. Nevertheless, the QE predicted by the model does not agree with the data. This discrepancy is addressed in the next section.

#### 3.3.3. Surface Passivation by Quantum Exclusion

The high QE and stability observed in delta-doped detectors is a consequence of the ultrathin surface passivation layer created by MBE growth. In the last two sections, we showed that the diffusion–drift QE model predicts moderate loss of signal due to surface recombination in delta-doped detectors, which is inconsistent with the near-100% IQE observed in delta-doped detectors (Figure 7). This discrepancy reflects a key limitation of the diffusion-drift QE model, which does not address quantum confinement and quantum transport of carriers in the MBE-grown silicon layer.

One essential difference between the diffusion–drift QE model and a fully quantum mechanical model of surface recombination is the non-local nature of electron and hole states in the delta-doped superlattice. In previous publications, we used software developed for semiconductor nanodevices to calculate quantized electron and hole states in delta-doped superlattices [23,24,25]. Calculations using nextnano++ [46] predict a surface barrier height of nearly 1 eV, which is significantly greater than the barrier height in Figure 8 because quantum confinement of majority carriers bound to the superlattice effectively increases the bandgap near the surface. In contrast, the wave functions of photogenerated electrons are unbound and non-localized, and have significant overlap with lower-energy states in bulk silicon on the detector side of the delta-doped superlattice. For this reason, minority carriers in the delta-doped superlattice can undergo quantum transport to either surface traps or bulk states in the detector, with asymmetric transition probabilities.

Stochastic models of charge transport in quantum systems may be applicable to calculations of QE in delta-doped detectors [47]. In these models, the QE and stability of a delta-doped detector would depend on the relative probabilities of capture by surface traps and quantum transitions to lower-energy, unbound states in the silicon detector. Based on QE measurements of delta-doped detectors, we can infer that these probabilities are highly asymmetric, with transitions to bulk states being far more likely than capture in surface traps. In this context, the observed QE and stability of delta-doped detectors are manifestations of an asymmetric exclusion process, which we have previously called surface passivation by quantum exclusion [24].

### 3.4. Limitations of the Model

A close study of Figure 1 and Figure 2 shows that, despite very good overall agreement, there are systematic deviations of the QE model from the data, which are informative, both for understanding the physics and for recognizing the limitations of the model and our imperfect knowledge of materials and device properties.

#### 3.4.1. Near Ultraviolet (200 to 380 nm)

Agreement between data and models in this region of the spectrum is generally good. The QE in this region is dominated by signal loss due to surface recombination, which is affected by the density of surface charge. Based on comparisons of QE data with models in Figure 1 and Figure 2, we estimated a surface charge density of approximately $4\times 10^{12}{\mathrm{cm}}^{-2}$ in the ion-implanted CCD characterized by Heymes et al. [40] and showed that the observed QE enhancement is consistent with UV-induced neutralization of oxide charge at a density of $10^{12}\;{\mathrm{cm}}^{-2}$.

#### 3.4.2. Far Ultraviolet (100 to 200 nm)

In this region of the spectrum, the measured QE is sensitive to the details of absorption and reflection in the surface oxide. The optical constants of native SiO_2_ are highly process-dependent and not well-known for the Heymes et al. detector [40]. In lieu of accurate data for the surface oxide, we have used optical constants for SiO_2_ from Palik, which is why the modeled QE shows a resonance near the SiO_2_ bandgap energy. The measured QE appears to show a resonance nearby, but the peak is shifted and dampened relative to the model. The surface oxide is so thin in this detector that variability in the oxide composition and bond angle near the interface are important. Extensive studies of the Si–SiO_2_ interface performed at JPL by Frank and Paula Grunthaner using X-ray photoelectron spectroscopy demonstrated significant variability of the bond angle and stoichiometry near the interface [48]. Studies of the optical properties of amorphous silica show that the bond angles vary from 136° to 180° and that these changes are found to correlate to changes in the bandgap energy from 8.4 to 11 eV [49].

#### 3.4.3. Extreme Ultraviolet (1 nm to 100 nm) and Visible (>380 nm)

In the extreme ultraviolet (1 nm<λ<100 nm), and correspondingly in the visible spectral range (λ>380 nm), photon absorption occurs primarily in the tail of the ion-implanted dopant distribution, overlapping very little with the surface depletion layer. In this spectral region, systematic variations between the model and the data may arise from incomplete knowledge of bandgap narrowing as a function of temperature.

Looking at Figure 2 in the spectral range from 3 nm to 10 nm, and Figure 1 from 380 nm to 400 nm, we see that the modeled QE is slightly higher than the measured QE. One possible explanation for this systematic deviation is recombination due to residual defects in the tail of the ion-implanted surface. Another possible explanation is a failure to account for temperature-dependent bandgap narrowing parameters in highly doped silicon. Whereas most of the empirical studies of bandgap narrowing in bipolar transistors have been carried out at higher temperatures (280 K to 400 K), to our knowledge only one paper has reported data on bandgap narrowing at cryogenic temperatures [50]. Unfortunately, the published data are not sufficiently comprehensive to support modeling of ion-implanted silicon detectors over the required range of dopant concentrations and temperatures.

At photon energies approaching the silicon K-edge (1 to 3 nm), the measured external QE approaches unity. In this spectral range, the photon absorption length varies from 0.3 to 2 µm, which is comparable to the EUV range from 12.5 to 30 nm, and the visible range from 450 to 600 nm. Data in these regions suggest that the IQE should be in the 90–95% range, with some loss of signal from photons absorbed near the surface. This discrepancy may be caused by a reduction in surface recombination due to the ballistic transport of hot electrons away from the surface.

#### 3.4.4. Soft X-ray (<1 nm)

At photon wavelengths shorter than 1 nm, we are entering the soft X-ray region of the spectrum. The QE data in Heymes et al. [40] are limited in this region, and the available data are dominated by the silicon K-edge at 1838.9 eV (wavelength ~ 0.7 nm). In this range, the photon absorption depth approaches the thickness of the detector, and the QE reaches a minimum due to losses through the front surface of the detector.

## 4. Discussion: Radiation-Induced Charging and Damage in Silicon Surfaces and Oxides

Radiation-induced charge injection and trap generation in silicon surfaces and oxides have important consequences for the stability of silicon detectors in space. In Section 3, we compared QE data and model calculations to derive quantitative estimates of the densities of surface traps and surface charge in radiation-damaged, ion-implanted and delta-doped CCDs and CMOS image sensors. The purpose of this section is to explore the microscopic processes underlying radiation damage in silicon detectors using data and theory describing the radiation-induced degradation of silicon metal-oxide-semiconductor (MOS) devices.

The microscopic processes involved in UV-induced surface charging of silicon devices are elucidated by a classic experiment performed at Caltech by Carver Mead in 1967. The experiments were designed to study UV annealing of oxide space charge created by X-ray damage to MOS structures. Measurements using UV photons at different energies led to the following conclusion: “It can be seen that there is a rather sharp threshold energy for annealing which is consistent with the Si–SiO_2_ barrier energy of 4.3 eV, indicating that electrons are injected from the silicon into the oxide conduction band, and subsequently neutralize the positive space charge” [51]. The same fundamental processes may well have caused UV-induced neutralization of positive charge in the CCDs characterized by Heymes et al. [40].

The UV-induced neutralization of oxide space charge provides a natural explanation for the observed saturation behavior in the UV-flood experiment reported in Heymes et al. The QE enhancement saturated after two hours, and then remained relatively stable during 15 h of additional exposure [40]. This saturation behavior is consistent with the dynamic model for hot carrier injection in MOS devices developed by Nissan-Cohen et al., in which the oxide charge reaches a steady-state trapping level that depends on the electric field in the oxide [52]. They went on to show that charge buildup in SiO_2_ is affected by two main mechanisms. On short time scales and at low injection rates, hot carrier injection leads to steady-state oxide charge, when a balance is achieved between opposing trapping and detrapping processes. On long time scales and high injection, charge buildup in the oxide is mainly due to the generation of new traps, and the subsequent partial occupation of the generated traps. On the microscopic mechanisms of trap generation, Nissan-Cohen wrote “The physical mechanism of radiation-induced trap generation can be related to the model of shallow traps in SiO_2_, which are commonly attributed to trivalent Si sites”. On the rates of trap generation: “The trap generation rate is found to be proportional to the flux of the injected charge, and to increase exponentially with the oxide electric field”. Radiation-induced trap densities can reach extraordinarily high values, approaching 10^20^ cm^−3^, while the trap occupation level depends strongly on the electric field [53].

Experimental studies of vacuum ultraviolet (VUV)-induced radiation damage in MOS oxides showed that whereas charge injection into thermal SiO_2_ is initially slow because of the small cross-section of traps in high-quality thermal oxides, ionizing radiation causes accelerated rates of charging and degradation due to “positive feedback in the generation of oxygen vacancies and the clustering of defects, which appear to take place in the degeneration of the MOS system upon VUV irradiation”. The radiation-induced destruction of the oxide network continues “without any indication that the process would saturate”, underscoring the importance of radiation-hardened surface passivation technologies for the stability and photometric accuracy of CCDs and CMOS image sensors in space [53].

## 5. Conclusions

In this study, we developed a model of QE in radiation-damaged detectors and compared the results with the performance of ion-implanted and delta-doped detectors measured before, during, and after illumination with intense ultraviolet light. Data and models presented in this paper elucidate the mechanisms and effects of surface charging and damage in silicon detectors. The importance of radiation-hardened surface passivation processes is highlighted by EUV-induced radiation damage in SOHO EIT CCDs [11,12,13]. Incomplete surface passivation in ion-implanted detectors leads to QE instabilities, especially after exposure to ionizing radiation increases the susceptibility to surface charging by generating interface and oxide traps in the detector surface. The QE of ion-implanted CCDs was found to be sensitive to variations in surface charge density as small as $10^{11}\;{\mathrm{cm}}^{-2}$, while delta-doped CCDs were found to be stable at surface charge densities as large as $10^{14}\;{\mathrm{cm}}^{-2}$.

An important premise of this paper is that surface damage caused by ultraviolet light is directly relevant to the performance of CCDs and CMOS image sensors in space. The significance of observed QE instabilities caused by ultraviolet light is twofold: (1) ultraviolet light induces surface charging by hot carrier injection and trapping of photogenerated charge; (2) ultraviolet light damages the detector surface by causing the formation of interface and oxide traps. In Section 4, we cited the scientific literature on the mechanisms of aging and degradation of MOS devices and structures to illuminate the microscopic processes responsible for the instabilities and degradation of CCDs and CMOS image sensors in space.

The physical and chemical reasons for the observed stability of delta-doped detectors were explored using QE measurements, model calculations, and references to prior work and the scientific literature on delta-doped surfaces. Previously published studies of delta-doped detectors show near-100% IQE and stable response to within 1%, despite UV-induced damage and variable surface charge at levels as high as 10^14^ cm^−2^. Counterintuitively, the diffusion-drift model links this stability to an exceptionally high density of charge trapped at the surface, corresponding to an effectively infinite surface recombination velocity in delta-doped detectors. Although the diffusion-drift model is consistent with the observed stability of delta-doped detectors, the modeled QE is inconsistent with the data.

The discrepancy between the measured and modeled QE of delta-doped detectors represents one of the key limitations of the diffusion–drift QE model developed in this paper. General considerations of quantization of electron and hole wave functions in the delta-doped superlattice suggest that the observed near-100% internal QE in delta-doped detectors can be explained by an asymmetric exclusion process, which we have previously called surface passivation by quantum exclusion [24]. The development of an improved QE model that incorporates the physics of quantum confinement and quantum transport in delta-doped surfaces is a challenge for future work.

CCDs and CMOS image sensors passivated with delta-doped superlattices are found to be uniquely stable against ionizing radiation damage, enabling the stability and photometric accuracy required by NASA for exoplanet science and time domain astronomy [54,55,56,57]. The high efficiency and stability of these detectors have been enabling in instrument and mission concept studies that are the basis for the Habitable Worlds Observatory, an ultra-stable, 6-m ultraviolet/optical/infrared telescope recommended by the National Academy of Sciences as the top priority for NASA’s Great Observatories Mission & Technology Maturation Program [1,58,59].

## Data Availability

Data are contained within the article.
